# Poly(vinyl alcohol boric acid)-Diclofenac Sodium Salt Drug Delivery Systems: Experimental and Theoretical Studies

**DOI:** 10.1155/2020/3124304

**Published:** 2020-05-31

**Authors:** Daniela Ailincai, Alexandra Maria Dorobanțu, Bogdan Dima, Ștefan Andrei Irimiciuc, Cristian Lupașcu, Maricel Agop, Orzan Olguta

**Affiliations:** ^1^Petru Poni Institute of Macromolecular Chemistry, Grigore Ghica Voda Alley, Iasi, Romania; ^2^Elias Emergency University Hospital, Dermatology Clinic, Bucharest, No. 17 Marasti Bvd., 011461 Bucharest, Romania; ^3^National Institute for Laser, Plasma & Radiation Physics, Magurele, Romania; ^4^Grigore T Popa University of Medicine and Pharmacy Iasi, St. Spiridon University Hospital, I Tanasescu VI Butureanu Surgery Clinic 1, Iasi, Romania; ^5^“Gheorghe Asachi” Techical University of Iasi, Department of Physics, D. Mangeron Bvd. No. 73, 700050 Iasi, Romania; ^6^Romanian Scientists Academy, 54 Splaiul Independentei Blvd., Bucharest 050094, Romania; ^7^Carol Davila University of Medicine and Pharmacy, Elias Emergency University Hospital, Dermatology Clinic, Bucharest, No. 17 Marasti Bvd., 011461 Bucharest, Romania

## Abstract

The main aim of the paper was to simulate the drug release by a multifractal theoretical model, as a valuable method to assess the drug release mechanism. To do this, drug delivery films were prepared by mixing poly(vinyl alcohol boric acid) (PVAB) and diclofenac (DCF) sodium salt drug in different mass ratios from 90/10 to 70/30, in order to obtain drug delivery systems with different releasing rates. The different drug content of the three systems was confirmed by energy-dispersive spectroscopy (EDAX) analysis, and the encapsulation particularities were investigated by scanning electron microscopy (SEM), atomic force microscopy (AFM), and polarized optical microscopy (POM) techniques. The ability of the PVAB matrix to anchor the DCF was assessed by Fourier transform infrared (FTIR) spectroscopy. The *in vitro* release of the diclofenac sodium salt from the formulations was investigated in biomimetic conditions (pH = 7.4 and 37°C) by UV-Vis spectroscopy, measuring the absorbance of the drug at 275 nm and fitting the results on a previously drawn calibration curve. An estimation of the drug release kinetics was performed by fitting three traditional mathematical models on experimental release data. Further, the drug delivery was simulated by the fractal theory of motion, in which the release dynamics of the polymer-drug complex system is described through various Riccati-type “regimes.” To explain such dynamics involved multifractal self-modulation in the form of period doubling, quasiperiodicity, intermittency, etc., as well as multifractal self-modulation of network type. Standard release dynamics were explained by multifractal behaviors of temporary kink type. The good correlation between the traditional mathematical models and the new proposed theoretical model demonstrated the validity of the multifractal model for the investigation of the drug release.

## 1. Introduction

Drug delivery is one of the most important fields related to medicine and healthcare, in which materials innovation plays a crucial role [1, 2]. Comprising different approaches and different systems, drug delivery is aimed at transporting pharmaceutical active compounds within a living organism, creating the frame for its therapeutic effect. The versatility and multitude of systems used in drug delivery comes from the wide range of both drugs and drugs carriers [3–8], considering that the same active molecule, encapsulated in a different carrier, gets a new life, due to the intrinsic properties of the carriers but also due to the interactions established between the matrix and the drug. Moreover, the existence of plenty of administration routes such as oral, nasal, ocular, and intravenous makes the field of drug delivery a complex one in which continuous development and innovation are required [9, 10].

Diclofenac (DCF) is a nonsteroid anti-inflammatory drug (NSAID), frequently used for the amelioration of acute or chronic painful conditions. It is efficient for the treatment of acute postoperative pain [11], osteoarthritis, rheumatoid arthritis, ankylosing spondylitis, extra-articular rheumatism [12], pancreatitis [13], urological disses [14], and so on. Moreover, recent evidences showed that DCF is also able to inhibit tumor angiogenesis of some cancer cell lines [15]. It appears that the common root of its anti-inflammatory and antitumor efficiency results from its ability to inhibit the cyclooxygenase enzyme [14]. DCF, which is a potent inhibitor of COX-2 and prostaglandin E2 synthesis, displays a range of effects on the immune system, angiogenic cascade, chemo- and radio-sensitivity, and tumor metabolism. Nevertheless, the inhibition of this enzyme was also associated with side effects, such as gastrointestinal disorders, liver hypersensitivity reaction, immune hemolytic anemia, and immune thrombocytopenia. Systematic investigations revealed that these side effects are caused by interplay of metabolic and immunologic factors, and their incidence is higher for the patients who take DCF at a dose of more than 150 mg/day [16]. Moreover, the low bioavailability of DCF reached by oral administration is a real concern, as its presence in environment raises health problems for aquatic organisms, plants, and mammals and led to the development of drug-resistant strains [17]. In this context, there is a constant concern to find new pathways for an efficient DCF administration, which should minimize the side effects. To this end, the design of formulations for DCF prolonged release keeps the promise of a potential solution. Many systems were designed and investigated, based on the encapsulation of DCF into a large realm of polymeric matrixes, based on carboxymethyl cellulose [18], silk fibroin membranes [19], chitosan [6], polyvinyl alcohol [20–23], poly(D,L-lactic acid-co-glycolic acid) [24], poly(D,L-lactide-co-glycolide) [25–28], poly(epsilon-capro/D,L-lactide) [29], poly(maleic anhydride-alt-2-methoxyethyl vinyl ether) [30, 31], pectin [32], and so on. These data showed that a prolonged release of diclofenac can be reached from polymeric matrices able to develop intermolecular forces with diclofenac molecules [6, 33]. With all these in mind, we designed novel formulations based on diclofenac and poly(vinyl alcohol boric acid) [4]. The rational design was raised from the idea of strong intermolecular forces between the electron-deficient boron atom of PVAB and esteric groups of diclofenac, which should assure a good anchoring of the drug into the matrix [34–37] and thus a slow release. The *in vitro* release has been investigated in an environment mimicking the body fluids, and the drug release kinetics was assessed by fitting the data on three traditional mathematical models. The next step in the investigation of such novel systems was the mathematical modeling of the diclofenac release by the fractal theory of motion, a new approach which offers various advantages when compared with the other well-known empirical and semiempirical models.

## 2. Materials and Methods

Diclofenac (DCF) sodium salt, polyvinyl alcohol boric acid (PVAB, Mw = 54000), and ethanol (>99.8%) were purchased from Sigma Aldrich and used without any previous purification, while double-distilled water was obtained in the laboratory.

### 2.1. Preparation of the Drug Delivery Systems

**Figure 1:**
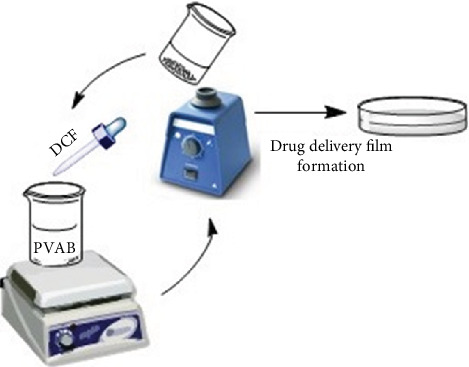
The obtaining of the drug delivery systems.

The drug delivery systems were obtained by solvent-induced phase separation method, by three consecutive cycles of 30 min stirring/5 min vortex applied to a mixture of PVAB and DCF solutions in different mass ratios [38, 39]. More precisely, to 10 mL PVAB solution 7.5% in water, were added 1.666, 3.75, and 6.42 mL solution of DCF in ethanol (5%), leading to three different formulations, D1, D2, and D3, containing different amounts of drugs. After the third cycle, the solutions were casted in Petri dishes and dried at room temperature, leading to self-standing flexible films (Figure 1).

### 2.2. The Evaluation of the DCF Release Kinetics

The ability of the obtained formulations to release the encapsulated drug was monitored *in vitro*, according to a previously described procedure [4]. Pieces of films from the D1-D3 formulations, containing the same amount of drug (14 mg), were used in the *in vitro* release determinations. The samples had sizes between ~1 cm^2^ and ~3 cm^2^ depending on drug content. They were immersed in 10 mL PBS, pH = 7.4. In order to mimic the *in vivo* conditions, the temperature was kept constant at 37°C all over the experiment. From time to time, 2 mL from the supernatant was removed and replaced with fresh PBS. The absorbance of the DCF drug in the supernatant was evaluated by UV-Vis spectroscopy and correlated with concentration through a previously drawn calibration curve, on the DCF absorption maximum from 275 nm [6]. All the measurements were done in triplicate.

The absorbance of the drug was measured on a PerkinElmer Lambda 35 UV-Vis spectrophotometer.

The kinetic data were fitted on three mathematical models, further described as follows:

- *Zero*-*Order Model*. *Q*_*t*_ = *K*_0_ · *t*, where *Q*_*t*_ is the amount of drug dissolved in the time *t* and *K*_0_ is the zero-order release constant
- *Higuchi Model*. *Q*_*t*_ = *K*_H_ · *t*^1/2^, where *Q*_*t*_ is the amount of drug released in the time *t* and *K*_H_ is the Higuchi dissolution constant
- *Korsmeyer-Peppas Model*. *M*_*t*_/*M*_∞_ = *K* · *t*^*n*^, where *M*_*t*_/*M*_∞_ is the fraction of drug released in the time *t*, *K* is the release rate constant, and *n* is the release exponent

### 2.3. Mathematical Model

#### 2.3.1. Release Dynamics through Multifractal Functions

Classical, commonly used models are usually founded on the otherwise unjustified supposition that variables describing the dynamics of any polymer-drug complex system are differentiable [40, 41] (for details, see the models of zero-order, Higuchi, Korsmeyer-Peppas, etc. [6]). Thus, the success of the abovementioned models should be understood as sequential, on domains in which differentiability and integrability are still valid. The differentiable and integrable mathematical procedures are otherwise inadequate when the dynamics of any polymer-drug complex system involves both nonlinearity and chaoticity. However, in order to describe such dynamics, but still employing differential mathematical procedures, it is necessary to introduce the scale resolution explicitly into the expression of physical variables and implicitly into the expression of fundamental equations that govern these dynamics. This means that any variable dependent on space and time coordinates, in a classical sense, will depend both on the space and time coordinates and on the scale resolutions in the new mathematical sense (that of nondifferentiability and nonintegrability). In other words, instead of operating with a variable described through a nondifferentiable function, we will work with approximations of this mathematical function, obtained by its mediation at various scale resolutions. As a result, any variable designed to describe the dynamics of any polymer-drug complex system will function as the limit of a family of mathematical functions, this being nondifferentiable for null scale resolution and differentiable for nonzero scale resolutions [42, 43].

This method of describing the dynamics of any polymer-drug complex system clearly implies the development of new geometrical structures and also of new mathematical models for which the motion laws, invariant to spatial and temporal transformations, are integrated with scale laws, invariant to spatial and temporal scale transformations. In our opinion, such a geometrical structure can be based on the concept of a “multifractal,” and the corresponding mathematical model can be based on the fractal theory of motion in an arbitrary and constant fractal dimension. Applications of the model in the analysis of the dynamics of complex systems are given in [44–53]. For complex systems with release, the dynamics analysis is given in [54–58].

The fundamental assumption of our model is the one that the dynamics of structural units of any polymer-drug complex system will be described by continuous but nondifferentiable motion curves (multifractal motion curves). These multifractal motion curves exhibit the property of self-similarity in every point, which can be translated into a property of holography (every part reflects the hole). Basically, we are discussing about “holographic implementations of structural units' dynamics of any polymer-drug complex system” through multifractal “regimes” of Riccati-type equations (describing the dynamics of structural units of any polymer-drug complex system by using Riccati-type equations at various scale resolutions).

Obviously, logistic-type equations (which are a particular case of Riccati-type equations) for describing the dynamics of the structural units of any complex polymer-drug system have been used [59]. However, their applicability is limited considering the absence of the scale resolution from the description of such dynamics. A first result within the meaning of the above was given by us in [60]. In what follows, we will analyze the dynamics in complex polymer-drug systems using Riccati-type equations at various scale resolutions (multifractal Riccati-type equations).

Let us consider now the multifractal Riccati-type equation:

(1) $$\begin{array}{c} A\frac{dM}{dt}=M^{2}-2BM-AC, \end{array}$$

where *M* is the multifractal mass of drug released at time *t* ≠ 0, *t* is the nonmultifractal time, with the role of affine parameter of the release curves, and *A*, *B*, and *C* are the multifractal parameters characteristic of the release dynamics.

These parameters depend on the intrinsic structure of the polymeric matrix and of the drug, on the polymer-drug matrix interactions, on the complex release system-biostructure interactions (which imply the immunity system of the biostructure), and on the scale resolution.

#### 2.3.2. Multifractal Transient Phenomena in Drug Delivery

With regard to the solution of the multifractal Riccati-type equation (1), we must first notice that the roots of the polynomial

(2) $$\begin{array}{c} P\left(M\right)=M^{2}-2BM-AC, \end{array}$$

can be written as

(3) $$\begin{array}{c} M_{1}\equiv B+iA\Omega ,\\ M_{2}\equiv B-iA\Omega ,\\ \;\Omega^{2}=\frac{C}{A}-{\left(\frac{B}{A}\right)}^{2},i=\sqrt{-1}. \end{array}$$

Performing the homographic transformation

(4) $$\begin{array}{c} Z=\frac{M-M_{1}}{M-M_{2}}, \end{array}$$

it results through direct calculus that *z* is a solution of the linear and homogenous first-order equation

(5) $$\begin{array}{c} z=2i\Omega z, \end{array}$$

which allows the solution

(6) $$\begin{array}{c} z\left(t\right)=z\left(0\right)e^{2i\Omega t}. \end{array}$$

**Figure 2:**
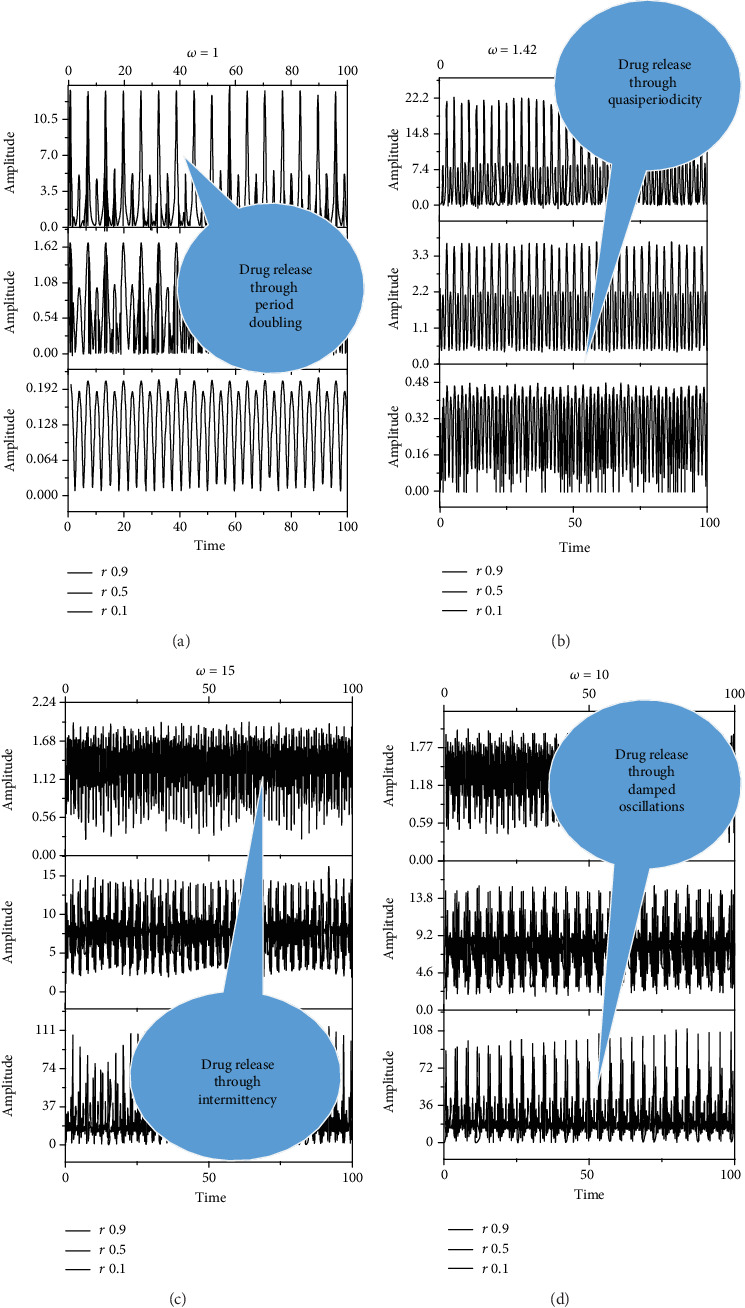
Dependences with time of *Rez* for different values of *ω* and *r*. (a) *ω* = 1; *r* = 0.1, 0.5, 0.9. (b) *ω* = 1.42; *r* = 0.1, 0.5, 0.9. (c) *ω* = 10; *r* = 0.1, 0.5, 0.9. (d) *ω* = 15; *r* = 0.1, 0.5, 0.9. Release dynamics through multifractal self-modulation at a nondifferentiable scale in the form of period doubling, quasiperiodicity, damped oscillations, and intermittency.

Therefore, if the initial condition *z*(0) is conveniently expressed, the general solution of (1) can be found by writing (4) as

(7) $$\begin{array}{c} M=\frac{M_{1}+{re}^{2i\Omega t}M_{2}}{1+{re}^{2i\Omega t}}, \end{array}$$

where *r* is a real multifractal constant which characterizes the solution. By using (3), we can write this solution in real terms, as

(8) $$\begin{array}{c} z=B+A\Omega \left(\frac{2r\;\sin\;2\Omega t}{1+r^{2}+2r\;\cos\;2\Omega t}+i\frac{1+r^{2}}{1+r^{2}+2r\;\cos\;2\Omega t}\right), \end{array}$$

which highlights a self-modulation of the pulsation-type characteristic *Ω* known as the Stoler-type transformation [61, 62] implying a complex form for this parameter. In Figure 2, we present this self-modulation phenomenon through *Rez* time dependences, for various values of *r* and *Ω*.

It results that the release dynamics are explained by multifractal self-modulation (at different scale resolutions given by the maximum values of (*ω*)) in the form of period doubling, quasiperiodicity, damped oscillations, intermittency, etc. (specificities dictated by the Fourier transforms of the analyzed quantities).

From a physical point of view, the fractal representation of the drug release mechanism showcased different release scenarios. These scenarios describe behaviors at a nondifferentiable (mesoscopic) scale from a local perspective encompassing all types of interactions between drug-polymer-medium. Our model predicts the drug release scenarios in the transition regime before the interactions reach an equilibrium drug release regime. In Figures 2(a) and 2(b), it can be seen a periodic release of specific quantities of the drug over a controllable period of time. The period doubling significance is important as each high concentration of drug is succeeded by a lower dose. In Figures 2(c) and 2(d), it can be seen that alternatives make these dynamics into more complex behaviors. It was observed that a useful damped periodical scenario do control the high doses of the specific drug. The intermittence release of drugs was based on a controllable modulating frequency (chosen as a control parameter). Finally, we achieved a modulated response of the drug release systems which can be controlled in both concentration and frequency of release. In the fractal paradigm, we can obtain various scenarios for the drug release mechanism, which help us to understand the behavior of drugs that may benefit from such complex scenarios.

**Figure 3:**
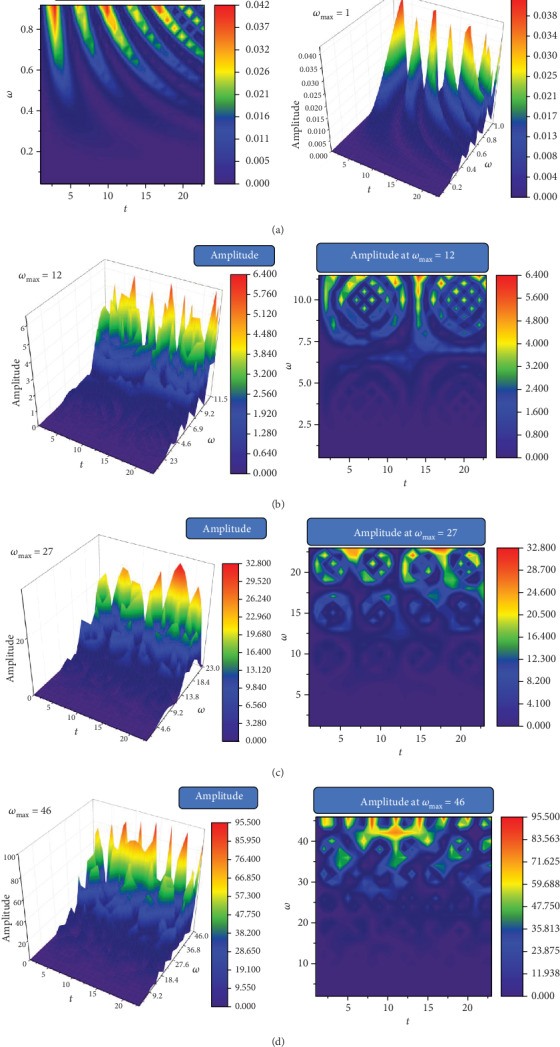
3D and 2D dependences of *Rez* for different values of *ω* and *t* at a constant value of (a) *ω* = 1, *r* = 0.5; (b) *ω* = 12, *r* = 0.5; (c) *ω* = 27, *r* = 0.5; and (d) *ω* = 46, *r* = 0.5.

The 3D and 2D (contour plot) dependences of *Rez* on *Ω* and *t*, for a constant value of *r*, are shown in Figure 3. In such a situation, the release dynamics involve multifractal self-modulation (at different nondifferentiable scale resolutions given by the maximum values of *ω*) of the network dynamics. The overall evolution of our system is clearly presented in Figure 3. There we can see the damped drug release scenario seen for various concentrations of the particular drug (controlled in our model by *ω*). For various nondifferentiable scale resolutions, we obtained different scenarios such as modulated release seen in Figure 3(b) while an intermittent release can be seen in Figure 3(c). The latter scenarios care complex release scenario and depend strongly on the physical properties of the polymer matrix and the biological release conditions. Finally, our model predicts the presence of unwanted regimes with a quasichaotic release. It is worth noticing that the complete chaotic dynamic is never reached, instead the adjustments made through the control parameter *ω* will force the system in a period doubling state. Therefore, even when the dynamic can seemingly be chaotic it can be easily rectified towards a more controllable state.

## 3. Results and Discussions

Three different formulations were obtained by combining a polymeric matrix-poly(vinyl alcohol boric acid) and diclofenac sodium salt anti-inflammatory drug, in different mass ratios from 70/30% to 90/10%. By casting the solutions into Petri dishes and slowly evaporating the water, self-standing flexible films were obtained, which were further characterized in terms of morphology, anchoring forces, and drug release rates.

### 3.1. Morphologic Characterization

**Figure 4:**
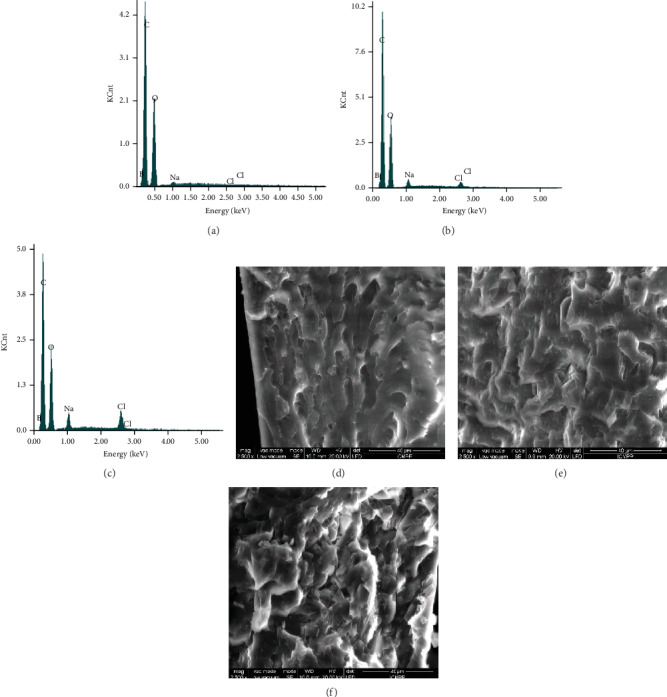
EDAX (a–c) and SEM (d–f) analysis on the D1-D3 films.

The EDAX analysis confirmed the obtaining of three drug delivery systems with different amounts of drug, by the increase of the percentage of sodium and chlorine starting from D1 to D3 (Figures 4(a)–4(c)). The corresponding SEM images indicated that DCF was homogenously encapsulated into the polymeric matrix as micrometric crystals, whose density depends on the mass ratio between PVAB polymer and DCF drug [39]. Therefore, in the case of the D1, which contains only 10% DCF, fewer micrometric geometric shapes were observed in comparison with the case of D3, in which the density was much higher (Figures 4(d)–4(f)).

**Figure 5:**
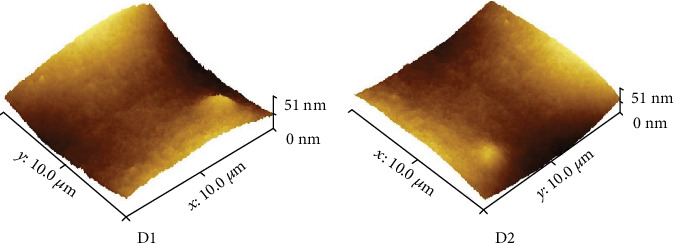
AFM images for representative drug delivery systems.

In order to have an insight about the morphology at nanoscale, the samples were further analyzed by AFM, which revealed the presence of nanograins in the case of all samples (Figure 5).

**Figure 6:**
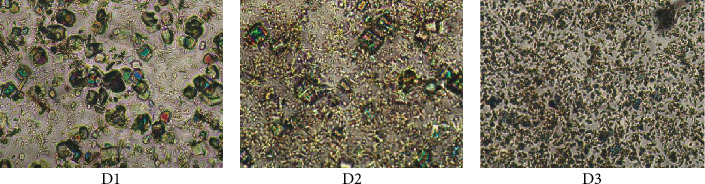
Polarized light microscopy images of the understudy drug delivery systems.

**Figure 7:**
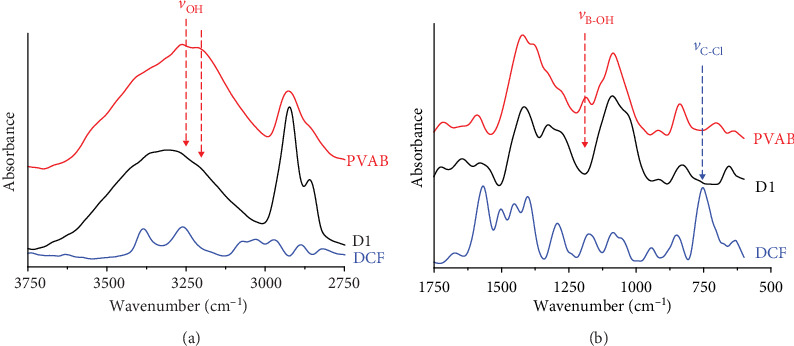
FTIR spectra of PVAB, DCF, and D1 formulation.

To confirm the crystalline nature of the encapsulated drug, the D1-D3 samples were analyzed by polarized light microscopy (Figure 6). As can be observed in Figure 7, the sample presented birefringent rectangular shapes characteristic to low molecular weight crystals [63], attributed to drug crystals, while the polymeric matrix did not present any birefringence, due to its amorphous nature. It can be observed that as the drug content increased, the size of the crystals decreased and their density increased, an aspect also seen for other systems based on PVAB matrix [34–36]. The explanation is related to the increased viscosity of the system containing a higher drug amount, due to the higher density of physical forces developed between electron-deficient PVAB matrix and DCF containing rich electron heteroatoms. The higher density hindered the diffusion of the DCF molecules and thus the growth of crystals.

### 3.2. Anchoring of the DCF into the PVAB Matrix

The PVAB polymer has been chosen as polymer matrix for DCF, anticipating strong interactions which should anchor the drug, prompting thus a prolonged release. In order to investigate them, the FTIR spectra were recorded for the formulations and for their components (Figure 6). In the spectra of both DCF and PVAB, multiple bands appeared in accordance with their structures as follows: both the DCF drug and PVAB polymer presented between 3750 and 2750 cm^−1^ two bands at 3386 and 3262 cm^−1^ in the case of DCF and a band with two maxima at 3277 and 3177 cm^−1^ in the case of the polymer, which correspond to NH and OH stretching vibrations, respectively [64]. In the fingerprint region, in the DCF spectrum, bands were observed corresponding to the stretching vibrations of the C-Cl bond at 743 cm^−1^ and vibrations of the carboxylic units at 1572 cm^−1^. The PVAB polymer presented also multiple peaks, the more significant ones being at 1330 and 1375 cm^−1^, due to the B-O bond stretching vibration and at ~1188 cm^−1^, due to B-OH deformation. In the spectra of the formulations, important changes appeared in both peak position and number. Therefore, in the 3750-2750 cm^−1^ spectral region, the two maxima from the polymer spectrum shifted to higher wavenumbers, indicating significant changes in the hydrogen bonds, indicating new interactions with the DCF molecules. The bands corresponding to B-OH deformation and C-Cl vibrations disappeared from the formulation spectrum indicating their implication in new strong interactions, the most probably coordinative bonds of the electron-deficient boron atom with electron-rich atoms of diclofenac (e.g. nitrogen, chloride). These spectral modifications clearly indicate that due to the structural peculiarities of both PVAB and DCF drug, strong interactions between them occurred in the formulations, the DCF molecules being “anchored” in the PVAB matrix by strong physical interactions, similar to liquid crystals previously reported by us [34–36].

### 3.3. The Release Kinetics

**Figure 8:**
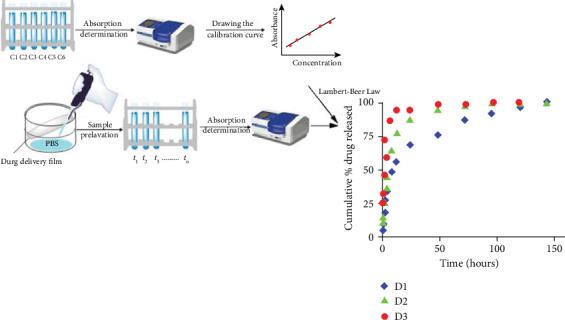
Representation of the protocol used for evaluating the release kinetics and cumulative % DCF release curves.

The release kinetics of the DCF drug from the formulations was investigated in biomimetic conditions, in PBS, at a pH of 7.4 and 37°C, according to a procedure previously described in the literature (Figure 8) [6].

As it could be observed, the samples released the encapsulated drug with different rates, which depend on the amount of DCF used in their preparation. Therefore, the D1 sample released the drug with a slower rate due to the fact that it contains only 10% drug in 90% PVAB polymer. This mass ratio between the components very probably led to a better dispersion and anchoring of the drug molecules in the polymeric matrix, influencing in a positive manner the drug release rate. When the amount of the drug increased, up to 30%, the releasing rate increased, leading to an increased burst effect. In all three cases, regardless the amount of the encapsulated drug, the release profile proceeded in two stages: in the first 4 hours, a burst effect was observed, while in the next hours (8-144 hours), the drug release rate decreased, leading to a sustained release (Figure 8, Table 1).

**Figure 9:**
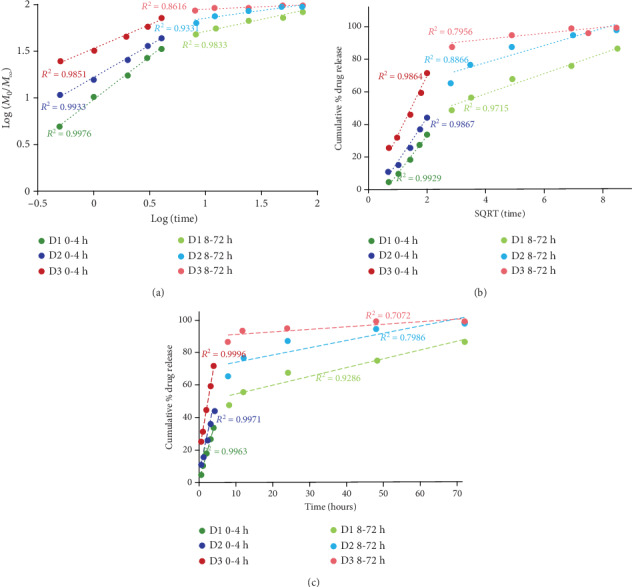
Linear forms of the Korsmeyer-Peppas (a), Higuchi (b), and zero-order (c) models applied for the release of DCF from the drug delivery systems for the two release stages.

The kinetic data obtained from the release experiments were fitted with three mathematical models as follows: zero-order, Higuchi, and Korsmeyer-Peppas, on both release stages (Figure 9). The high values obtained for the correlation coefficient (>0.98) in the first stage for the three mathematical models revealed that the DCF release from the drug delivery systems was a process controlled mainly by two factors—drug dissolution and its diffusion through the polymeric matrix. In the second stage, the kinetic models fitted pretty well only for the D1 sample. This indicates that the drug release was mainly governed by the matrix erosion in the second-stage process which was also confirmed with the naked eye.

To assess the performances of the PVAB matrix for drug encapsulation, compared to other polymers, we realized a literature survey on other drug delivery systems based on biopolymers and DCF. As can be seen in Table 2, PVAB has the ability to encapsulate bigger amounts of drug, which were released in a more prolonged manner. This confirmed that the PVAB ability to strongly anchor DCF is beneficial to encapsulate a high amount of drugs and further to favor its prolonged release.

In our mathematical model, the drug release mechanism was developed in the framework of the fractal theory of motion in which dynamics were described by means of fractal functions. More precisely, the various interactions of the polymer-drug-medium as a complex system are described through various Riccati-type “regimes” (multifractal Riccati-type equations). We obtained several release kinetic scenarios at a nondifferentiable (mesoscopic) scale. These scenarios mimic the usual chaos transition scenarios: period doubling, quasiperiodicity, damped oscillations, intermittency, and quasichaoticity. Moreover, we can find specific oscillation modes of the complex polymer-drug networks, where the kink-type solution corresponds to a steady macroscopic behavior. The kink solution will be used to validate the above-presented experimental data.

Indeed, the multifractal Riccati-type equation (1) has the bounded solution:

(9) $$\begin{array}{c} \frac{A}{{\left(AC+B^{2}\right)}^{1/2}}\tan h^{\left(-1\right)}\frac{M-B}{{\left(AC+B^{2}\right)}^{1/2}}=t. \end{array}$$

**Figure 10:**
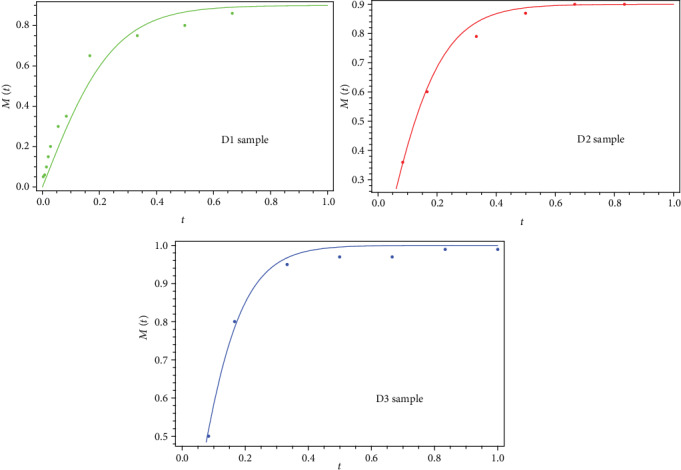
Correl ations b etween the theoretical (lines) and experimental data (points) obtained for *B* = 0, *a* = 0.9, and *A* = 0.225 (D1 sample); *B* = 0, *a* = 0.9, and *A* = 0.18 (D2 sample); and *B* = 0, *a* = 0.9, and *A* = 0.15 (D3 sample).

If we set ≡(*AC* + *B*^2^)^1/2^, then

(10) $$\begin{array}{c} M\left(t\right)=B+a\cdot \tan h\left(\frac{at}{A}\right), \end{array}$$

a relation that can describe the standard dynamics of release at various differentiable scale resolutions, through a convenient choice of variables and parameters (Figure 10). It results that standard release dynamics are explained by temporary kink-type multifractal behaviors (for details on nonlinear kink solutions, see [67–69]). We notice also that such a particular solution of our model fits well the experimental data. These results underline the generality of the fractal approach and its wide range in terms of drug release scenarios, strongly depending on the physical properties of the polymer matrix and medium properties.

## 4. Conclusions

A series of formulations based on poly(vinyl alcohol boric acid) and diclofenac sodium salt were prepared, and the drug release kinetics was investigated by three traditional mathematical models and a new theoretical multifractal model proposed by us. The drug was encapsulated into the polymeric matrix as micrometric and submicrometric crystals, anchored by physical forces. This assured a prolonged release of the DCF in two stages, reaching a period of 5 days in the case of the sample which contained the 10% drug. The fitting of the three traditional mathematical models on experimental release data suggested that drug release was governed by the DCF dissolution and diffusion in the first stage (four hours) and by the matrix erosion in the second one (five days). The modeling of the drug release by the fractal theory of motion evidenced several transient scenarios for a nondifferentiable (mesoscopic) scale in the form of period doubling, quasiperiodicity, damped oscillations, intermittency, and quasichaoticity. Usually, such behaviors were assimilated to the chaos transition scenarios of the complex system dynamics. These scenarios are specific oscillation modes of complex polymer-drug networks, where the kink-type solution corresponds to a steady macroscopic behavior. In such a situation, through a convenient choice of variables and release parameters, the theoretical model was validated on the basis of experimental data.

## Figures and Tables

**Table 1:** The mean values of the cumulative % DCF released ± standard deviation for 3 independent determinations.

Time of determination (hours)	Cumulative % drug released ± S.D.		
	D1	D2	D3
0.5	4.978 ± 0.0100	10.869 ± 0.0073	25.198 ± 0.0316
1	10.343 ± 0.0479	15.830 ± 0.0272	32.120 ± 0.0206
2	18.389 ± 0.0520	25.746 ± 0.016	45.690 ± 0.0247
3	27.183 ± 0.0502	36.911 ± 0.0120	59.425 ± 0.0080
4	33.883 ± 0.0731	44.679 ± 0.0324	71.856 ± 0.0415
8	48.665 ± 0.1219	65.842 ± 0.0341	86.947 ± 0.0748
12	56.430 ± 0.1087	77.017 ± 0.0516	93.719 ± 0.0711
24	68.345 ± 0.1128	87.508 ± 0.0415	95.177 ± 0.0784
48	75.825 ± 0.1322	94.804 ± 0.0523	99.161 ± 0.0644
72	86.543 ± 0.1052	97.692 ± 0.0478	99.336 ± 0.0452
96	91.948 ± 0.2679	99.153 ± 0.0706	99.9199 ± 0.0774
120	96.597 ± 0.2562	99.512 ± 0.0609	99.990 ± 0.0943
144	99.779 ± 0.2333	99.548 ± 0.0749	99.990 ± 0.0943

**Table 2:** Drug release from various formulations.

System	Drug content	Cumulative DCF release at 4 hours	Cumulative DCF release at 8 hours	Cumulative DCF release at 24 hours	Reference
PVAB film	10%	33	48	68	This study
	30%	71	86	95	
Methyl cellulose film	0.3%	45	85	ud	[65]
Sodium methyl cellulose film	0.3%	40	>80	ud	
Sodium alginate films	—	>97%	ud	ud	[22]
Sodium alginate/hydroxypropyl cellulose film	—	>99%	ud	ud	
Sodium alginate/hydroxypropyl cellulose film treated with CaCl_2_	—	53.25%	ud	ud	
Hydroxypropyl methylcellulose/polyvinyl pyrrolidone film	3.2%	99.7%	ud	ud	[66]
Poly(D,L-lactic acid-co-glycolic acid)/poly(ethylene glycol) scaffolds	—	55–62%	ud	75-82%	[24]
Chitosan/PVA cross-linked tripolyphosphate sodium	—	42%	78%	ud	[21]

## Data Availability

The data used to support the findings of this study are available from the corresponding author upon request.

## References

[1] Kakkar A., Traverso G., Farokhzad O. C., Weissleder R., Langer R. (2017). Evolution of macromolecular complexity in drug delivery systems. *Nature Reviews Chemistry*.

[2] Tiwari G., Tiwari R., Bannerjee S. K. (2012). Drug delivery systems: An updated review. *International Journal of Pharmaceutical Investigation*.

[3] Jacob J., Haponiuk J. T., Thomas S., Gopi S. (2018). Biopolymer based nanomaterials in drug delivery systems: A review. *Materials Today Chemistry*.

[4] Ailincai D., Gavril G., Marin L. (2020). Polyvinyl alcohol boric acid – A promising tool for the development of sustained release drug delivery systems. *Materials Science and Engineering: C*.

[5] Ailincai D., Mititelu L. T., Marin L. (2018). Drug delivery systems based on biocompatible imino-chitosan hydrogels for local anticancer therapy. *Drug Delivery*.

[6] Craciun A. M., Mititelu Tartau L., Pinteala M., Marin L. (2019). Nitrosalicyl-imine-chitosan hydrogels based drug delivery systems for long term sustained release in local therapy. *Journal of Colloid and Interface Science*.

[7] Olaru A. M., Marin L., Morariu S., Pricope G., Pinteala M., Tartau-Mititelu L. (2018). Biocompatible chitosan based hydrogels for potential application in local tumour therapy. *Carbohydrate Polymers*.

[8] Ailincai D., Marin L., Morariu S. (2016). Dual crosslinked iminoboronate-chitosan hydrogels with strong antifungal activity against Candida planktonic yeasts and biofilms. *Carbohydrate Polymers*.

[9] Prausnitz M. R., Langer R. (2008). Transdermal drug delivery. *Nature Biotechnology*.

[10] Patel A., Cholkar K., Agrahari V., Mitra A. K. (2013). Ocular drug delivery systems: an overview. *World Journal of Pharmacology*.

[11] Moore R. A., Derry S. (2018). Diclofenac Potassium in Acute Postoperative Pain and Dysmenorrhoea: Results from Comprehensive Clinical Trial Reports. *Pain Research and Management*.

[12] Atzeni F., Masala I. F., Sarzi-Puttini P. (2018). A review of chronic musculoskeletal pain: central and peripheral effects of Diclofenac. *Pain and Therapy*.

[13] Kumar N. S., Muktesh G., Samra T. (2020). Comparison of efficacy of diclofenac and tramadol in relieving pain in patients of acute pancreatitis: A randomized parallel group double blind active controlled pilot study. *European Journal of Pain*.

[14] Ulubay M., Yurt K. K., Kaplan A. A., Atilla M. K. (2018). The use of diclofenac sodium in urological practice: a structural and neurochemical based review. *Journal of Chemical Neuroanatomy*.

[15] Pantziarka P., Sukhatme V., Bouche G., Melhuis L., Sukhatme V. P. (2016). Repurposing Drugs in Oncology (ReDO)—diclofenac as an anti-cancer agent. *Ecancermedicalscience*.

[16] Villalonga N., David M., Bielanska J. (2010). Immunomodulatory effects of diclofenac in leukocytes through the targeting of Kv1.3 voltage-dependent potassium channels. *Biochemical Pharmacology*.

[17] Sathishkumar P., Meena R. A. A., Palanisami T., Ashokkumar V., Palvannan T., Gu F. L. (2020). Occurrence, interactive effects and ecological risk of diclofenac in environmental compartments and biota - a review. *Science of The Total Environment*.

[18] Maver U., Xhanari K., Žižek M. (2020). Carboxymethyl cellulose/diclofenac bioactive coatings on AISI 316LVM for controlled drug delivery, and improved osteogenic potential. *Carbohydrate Polymers*.

[19] Tomoda B. T., Corazza F. G., Beppu M. M., Lopes P. S., Moraes M. A. (2019). Silk fibroin membranes with self-assembled globular structures for controlled drug release. *Journal of Applied Polymer Science*.

[20] Saadon S., Razak S. I. A., Ismail A. E., Nayan N. H. M., Fakhruddin K. (2019). Influence of diclofenac sodium loading on physicochemical and mechanical properties of dual layer polyvinyl alcohol transdermal patch. *Journal of Physics: Conference Series*.

[21] Reveny J., Sumaiyah S. (2018). Formulation and evaluation of in vitro transdermal patch diclofenac sodium using chitosan polymer and polyvinyl alcohol cross-linked tripolyphosphate Sodium. *Asian Journal of Pharmaceutical and Clinical Research*.

[22] Sanlı O., Ay N., Isıklan N. (2007). Release characteristics of diclofenac sodium from poly(vinyl alcohol)/sodium alginate and poly(vinyl alcohol)-grafted-poly(acrylamide)/sodium alginate blend beads. *European Journal of Pharmaceutics and Biopharmaceutics*.

[23] Kharaghani D., Gitigard P., Ohtani H. (2019). Design and characterization of dual drug delivery based on *in-situ* assembled PVA/PAN core-shell nanofibers for wound dressing application. *Scientific Reports*.

[24] Sidney L. E., Heathman T. R. J., Britchford E. R., Abed A., Rahman C. V., Buttery L. D. K. (2015). Investigation of Localized Delivery of Diclofenac Sodium from Poly(D,L-Lactic Acid-*co*-Glycolic Acid)/Poly(Ethylene Glycol) Scaffolds Using an *In Vitro* Osteoblast Inflammation Model. *Tissue Engineering Part A*.

[25] Will O. M., Purcz N., Chalaris A. (2016). Increased survival rate by local release of diclofenac in a murine model of recurrent oral carcinoma. *International Journal of Nanomedicine*.

[26] Nikkola L., Morton T., Balmayor E. R. (2015). Fabrication of electrospun poly(D,L lactide-co-glycolide)80/20 scaffolds loaded with diclofenac sodium for tissue engineering. *European Journal of Medical Research*.

[27] Nikkola L., Viitanen P., Ashammakhi N. (2009). Temporal control of drug release from biodegradable polymer: Multicomponent diclofenac sodium releasing PLGA 80/20 rod. *Journal of Biomedical Materials Research Part B: Applied Biomaterials*.

[28] Viitanen P., Suokas E., Törmälä P., Ashammakhi N. (2006). Release of diclofenac sodium from polylactide-co-glycolide 80/20 rods. *Journal of Materials Science: Materials in Medicine*.

[29] Nikkola L., Seppälä J., Harlin A., Ndreu A., Ashammakhi N. (2006). Electrospun multifunctional diclofenac sodium releasing nanoscaffold. *Journal of Nanoscience and Nanotechnology*.

[30] Piras A. M., Chiellini F., Chiellini E., Nikkola L., Ashammakhi N. (2008). New multicomponent bioerodible electrospun nanofibers for dual-controlled drug release. *Journal of Bioactive and Compatible Polymers*.

[31] Piras A. M., Nikkola L., Chiellini F., Ashammakhi N., Chiellini E. (2006). Development of diclofenac sodium releasing bio-erodible polymeric nanomats. *Journal of Nanoscience and Nanotechnology*.

[32] Wang S. Y., Li J., Zhou Y., Li D. Q., Du G. M. (2019). Chemical cross-linking approach for prolonging diclofenac sodium release from pectin-based delivery system. *International Journal of Biological Macromolecules*.

[33] Pygall S. R., Griffiths P. C., Wolf B., Timmins P., Melia C. D. (2011). Solution interactions of diclofenac sodium and meclofenamic acid sodium with hydroxypropyl methylcellulose (HPMC). *International Journal of Pharmaceutics*.

[34] Marin L., Ailincai D., Paslaru E. (2014). Monodisperse PDLC composites generated by use of polyvinyl alcohol boric acid as matrix. *RSC Advances*.

[35] Ailincai D., Farcau C., Paslaru E., Marin L. (2016). PDLC composites based on polyvinyl boric acid matrix–a promising pathway towards biomedical engineering. *Liquid Crystals*.

[36] Ailincai D., Pamfil D., Marin L. (2018). Multiple bio-responsive polymer dispersed liquid crystal composites for sensing applications. *Journal of Molecular Liquids*.

[37] Ailincai D., Marin L., Shova S., Tuchilus C. (2016). Benzoate liquid crystals with direct isotropic–smectic transition and antipathogenic activity. *Comptes Rendus Chimie*.

[38] Marin L., Popescu M. C., Zabulica A., Uji-I H., Fron E. (2013). Chitosan as matrix for bio-polymer dispersed liquid crystal systems. *Carbohydrate Polymers*.

[39] Perju E., Marin L., Grigoras V. C., Bruma M. (2011). Thermotropic and optical behaviour of new PDLC systems based on a polysulfone matrix and a cyanoazomethine liquid crystal. *Liquid Crystals*.

[40] Mirchell M. (2009). *Complexity: A Guided Tour*.

[41] Badii R., Politi A. (2009). *Complexity: Hierarchical Structure and Scaling in Physics*.

[42] Mandelbrot B. B., Wheeler J. A. (1983). The Fractal Geometry of Nature. *American Journal of Physics*.

[43] Nottale L. (2011). *Scale Relativity and Fractal Space-Time- A New Approach to Unifying Relativity and Quantum Mechanics*.

[44] Gottlieb I., Agop M., Ciobanu G., Stroe A. (2006). El Naschie’s *ε*(∞) space–time and new results in scale relativity theories. *Chaos, Solitons & Fractals*.

[45] Agop M., Nica P., Ioannou P. D., Malandraki O., Gavanas-Pahomi I. (2007). El Naschie’s *ε*(∞) space–time, hydrodynamic model of scale relativity theory and some applications. *Chaos, Solitons & Fractals*.

[46] Nedeff V., Moşneguţu E., Panainte M. (2012). Dynamics in the boundary layer of a flat particle. *Powder Technology*.

[47] Agop M., Nica P., Gîrţu M. (2008). On the vacuum status in Weyl–Dirac theory. *General Relativity and Gravitation*.

[48] Gottlieb I., Agop M., Jarcǎu M. (2004). El Naschie’s Cantorian space–time and general relativity by means of Barbilian’s group.: A Cantorian fractal axiomatic model of space–time. *Chaos, Solitons & Fractals*.

[49] Agop M., Ioannou P., Nica P., Radu C., Alexandru A., Vizureanu P. (2004). Fractal Characteristics of the Solidification Process. *Materials Transactions*.

[50] Agop M., Griga V., Ciobanu B. (1998). Gravity and Cantorian space-time. *Chaos, Solitons & Fractals*.

[51] Ciubotariu C., Agop M. (1996). Absence of a gravitational analog to the Meissner effect. *General Relativity and Gravitation*.

[52] Niculescu O., Dimitriu D. G., Paun V. P., Matasaru P. D., Scurtu D., Agop M. (2010). Experimental and theoretical investigations of a plasma fireball dynamics. *Physics of Plasmas*.

[53] Agop M., Nica P. E., Gurlui S., Focsa C., Paun V. P., Colotin M. (2010). Implications of an extended fractal hydrodynamic model. *The European Physical Journal D*.

[54] Bacaita E. S., Agop M. (2016). A multiscale mechanism of drug release from polymeric matrices: confirmation through a nonlinear theoretical model. *Physical Chemistry Chemical Physics*.

[55] Balaita L., Chailanb J. F., Nguyen X. H., Bacaita S., Popa M. (2014). Hybrid Chitosan-Gelatine magnetic polymer particles for drug release. *Journal Of Optoelectronics And Advanced Materials*.

[56] Durdureanu-Angheluta A., Băcăiţă S., Radu V. (2013). Mathematical modelling of the release profile of anthraquinone-derived drugs encapsulated on magnetite nanoparticles. *Revue Roumaine de Chimie*.

[57] Radu V., Băcăiţă S., Uliniuc A., Popa M., Susanu S. (2013). Fractal hydrodynamic model for drug release processes from starch based hydrogels. *Materiale Plastice*.

[58] Magop D., Băcăiţă S., Peptu C., Popa M., Agop M. (2012). Non-differentiability at mesoscopic scale in drug release processes from polymer microparticles. *Materiale Plastice*.

[59] Ghosal K., Chandra A., Rajabalaya R., Chakraborty S., Nanda A. (2012). Mathematical modeling of drug release profiles for modified hydrophobic HPMC based gels. *Pharmazie*.

[60] Craciun A. M., Barhalescu M. L., Agop M., Ochiuz L. (2019). Theoretical Modeling of Long-Time Drug Release from Nitrosalicyl-Imine- Chitosan Hydrogels through Multifractal Logistic Type Laws. *Computational and Mathematical Methods in Medicine*.

[61] Stoler D. (1970). Equivalence classes of minimum uncertainty packets. *Physical Review D*.

[62] Stoler D. (1971). Generalized coherent states. *Physical Review D*.

[63] Zabulica A., Balan M., Belei D., Sava M., Simionescu B. C., Marin L. (2013). Novel luminescent phenothiazine-based Schiff bases with tuned morphology. Synthesis, structure, photophysical and thermotropic characterization. *Dyes and Pigments*.

[64] Marin L., Moraru S., Popescu M.-C. (2014). Out-of-water constitutional self-organization of chitosan–cinnamaldehyde dynagels. *Chemistry - A European Journal*.

[65] Ghosal K., Das A., Das S. K., Mahmood S., Ramadan M. A. M., Thomas S. (2019). Synthesis and characterization of interpenetrating polymeric networks based bio-composite alginate film: A well-designed drug delivery platform. *International Journal of Biological Macromolecules*.

[66] Jafariazar Z., Jamalinia N., Ghorbani-Bidkorbeh F., Mortazavi S. A. (2015). Design and Evaluation of Ocular Controlled Delivery System for Sodium. *Iranian journal of pharmaceutical research:IJPR*.

[67] Mazilu N., Agop M., Gatu I., Iacob D. D., Butuc I., Ghizdovat V. (2016). The classical theory of light colors: a paradigm for description of particle interactions. *International Journal of Theoretical Physics*.

[68] Mazilu N., Agop M., Gatu I., Iacob D. D., Ghizdovăt V. (2016). From Kepler problem to skyrmions. *Modern Physics Letters B*.

[69] Mazilu N., Ghizdovat V., Agop M. (2016). Role of surface gauging in extended particle interactions: The case for spin. *The European Physical Journal Plus*.

